# The influence of storage time and temperature on propofol concentrations in canine blood and plasma

**DOI:** 10.7717/peerj.3476

**Published:** 2017-06-30

**Authors:** Sherry Cox, Joan Bailey, Chika Okafor, Reza Seddighi, Tom Doherty

**Affiliations:** 1 Department of Biomedical and Diagnostic Sciences, University of Tennessee, Knoxville, TN, United States of America; 2 Department of Large Animal Clinical Sciences, University of Tennessee, Knoxville, TN, United States of America

**Keywords:** Propofol, Stability, Blood, Plasma, Temperature

## Abstract

Propofol is an intravenous anesthetic commonly used due to its favorable pharmacokinetic and pharmacodynamic profile. There are discrepancies in the literature about the most appropriate sample for determining propofol concentrations. Although plasma has been used for determining propofol concentrations, whole blood has been the preferred sample. There is also a lack of consistency in the literature on the effect of storage time and temperature on propofol concentrations and this may lead to errors in the design of pharmacokinetic/pharmacodynamics studies. The purpose of this study was to determine the difference in propofol concentrations in whole blood versus plasma and to evaluate the influence of storage time (56 days) and temperature (4 °C, −20 °C, −80 °C) on the stability of propofol concentrations in blood and plasma samples. Results from the study indicate that whole blood and plasma samples containing propofol stored at −80 °C have concentrations as high as or higher than those stored at 4 °C or −20 °C for 56 days; thus, −80 °C is an appropriate temperature for propofol sample storage. Plasma propofol concentrations were consistently higher than whole blood for all three storage temperatures. Consequently, plasma is the most appropriate sample for propofol analysis due to its consistent determinations.

## Introduction

Propofol is a short-acting intravenous anesthetic, which is associated with smooth and rapid inductions and recovery and is commonly used in dogs and other veterinary patients (Robertson, Johnston & Beemsterboer, 1992; Zoran, Riedesel & Dyer, 1993; Mandsager et al., 1995; Lee et al., 2012; Zonca et al., 2012; Miryabe-Nishiwake et al., 2013; Gomulka et al., 2015; Cattai et al., 2016). Propofol is weakly acidic, and drugs of this type are generally considered to bind to albumin in plasma. It is also a lipophilic drug, and despite being highly (98%) bound to serum/plasma proteins (Servin et al., 1988; Campos et al., 2016), it is approximately 50% bound to erythrocytes (Mazoit & Samili, 1999). Data from pharmacokinetic/pharmacodynamics studies based on the relationship between blood propofol concentrations and its effects have been used to design propofol dosage regimens for anesthesia (Cuadrado et al., 1998); however, differences in measured propofol concentrations due to the effects of storage time and temperature on plasma and whole blood samples may influence the dosage regimen design. Blood has been the medium of choice for determining propofol concentrations (Adam et al., 1981; Plummer, 1987; Chan & So, 1990; Zonca et al., 2012; Cattai et al., 2016). However, plasma (or serum) has been used for pharmacokinetic and pharmacodynamics studies, information comparing propofol concentrations in those fluids with whole blood is scarce: in some studies, a plasma/blood ratio of one or less has been described; therefore, propofol may be equally distributed between plasma and blood (Servin et al., 1988; Coetzee et al., 1995; Cuadrado et al., 1998). Whether plasma concentrations reflect the effects of propofol in humans and animals better than whole blood concentrations also remains to be established. Consequently, there is discussion in the literature as to whether propofol concentrations should be determined in whole blood or serum/plasma samples.

The method used to store samples may influence the concentration of drugs. It has been suggested that blood samples used for propofol determination should not be frozen (Plummer, 1987), and that propofol concentrations in samples stored at 4 °C are stable for up to one week (Adam et al., 1981; Zonca et al., 2012), two weeks (Cuadrado et al., 1998) or 12 weeks (Plummer, 1987). Bienert et al. (2005) suggested that blood should be stored at 4 °C but not at −20 °C because of significant propofol loss, however, they recommend that samples should be analyzed as soon as possible. They also suggest that plasma samples are stable at 4 °C for 60 days, and plasma provides a better matrix for propofol analysis.

The pharmacokinetics of propofol have been widely investigated, usually by determination of propofol in whole blood by the use of high performance liquid chromatography. The authors’ laboratory started analyzing propofol samples in 2009 and, at that time, whole blood analysis for propofol seemed to be the most appropriate method because of its interaction with erythrocytes. However, due to recent discussions in the literature it was felt that a re-evaluation of the sample matrix for propofol analysis was warranted. Additionally, samples are stored −80 °C in the laboratory and presently there are no data in the literature about sample stability at this temperature. Thus, the purpose of this study was to determine the effect of storage duration and temperature on stability of propofol concentrations in blood and plasma and determine if blood or plasma is an appropriate sample matrix. To achieve the objectives of this study, the following three specific aims were pursued: (1) compared the stability of propofol concentrations between blood and plasma samples. It was hypothesized that plasma is an acceptable sample for propofol studies. (2) Determined the stability of propofol concentrations at 4 °C, −20 °C, and −80 °C storage temperatures in blood and plasma. The working hypothesis was that −80 °C is an acceptable storage temperature for propofol studies. (3) Determined the stability of propofol concentrations at various storage periods (Day 1, 7, 14, 21, 28, 35, 42, 49 and 56) in blood and plasma. It was hypothesized that the stability of propofol would decrease with increased storage duration.

## Materials and Methods

### Equipment

Propofol was separated on a Waters XBridge C_18_ (4.6 × 250 mm, 5 µm) column with an XBridge C_18_ guard column. The mobile phase was a mixture of (A) water adjusted to pH 4.0 with glacial acetic acid and (B) acetonitrile (31:69). The flow rate was 1.5 mL/min and the column temperature ambient (24 °C). The fluorescence detector was set at an excitation of 276 nm and an emission of 310 nm with the gain at 10×.

**Figure 1:**
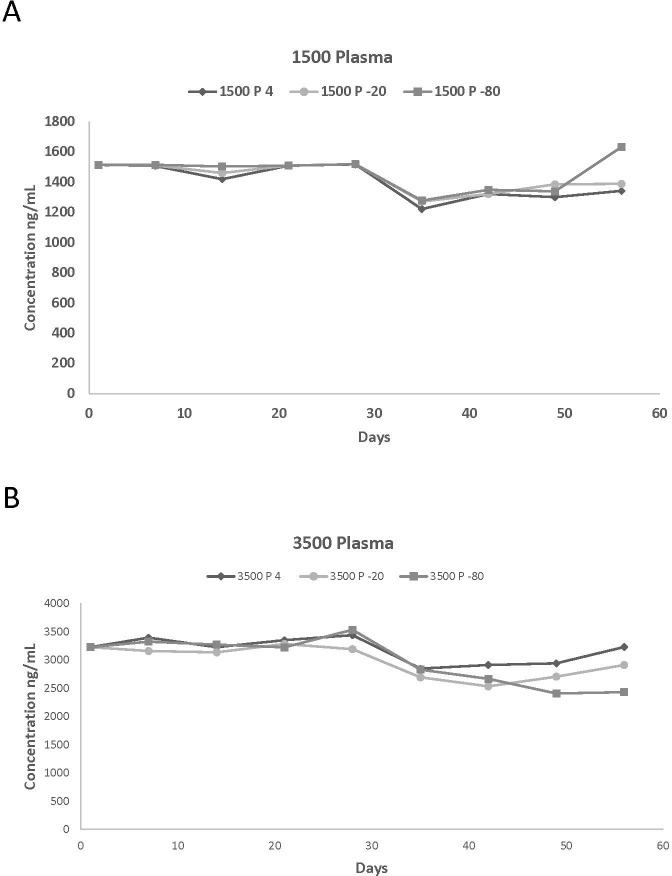
Plasma concentrations spiked with (A) 1,500 and (B) 3,500 ng/mL of propofol stored at 4 °C, 20 °C, and −80 °C for 56 days. The number of replicates per day is one.

**Figure 2:**
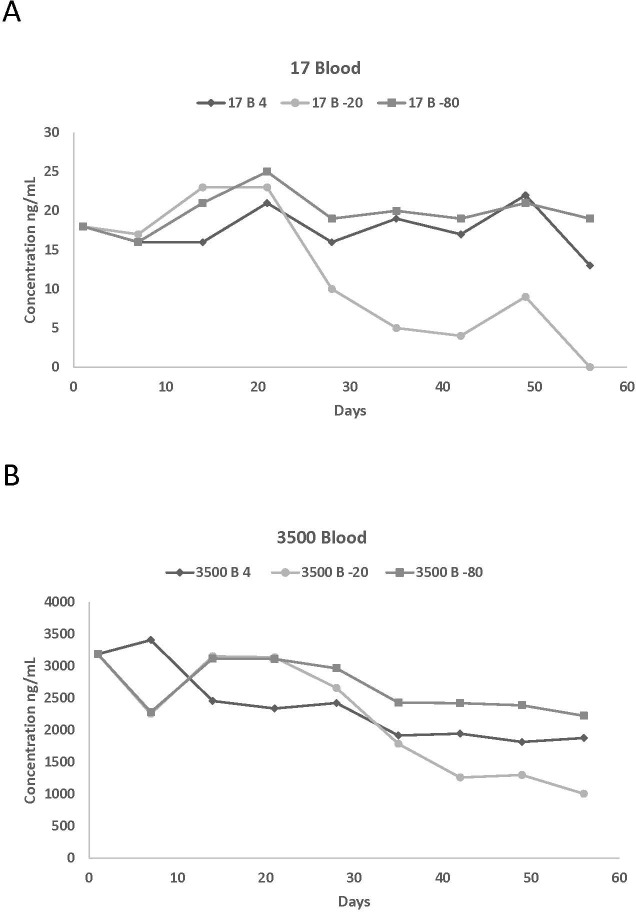
Blood concentrations spiked with (A) 17 and (B) 3,500 ng/mL of propofol stored at 4 °C, 20 °C, and −80 °C for 56 days. The number of replicates per day is one.

**Table 1:** Propofol plasma concentrations measured for 56 days at 4 °C, −20 °C, and −80 °C.

Temp	Concentration at different days of storage								
Starting conc.	Day 1^*^	Day 7	Day 14	Day 21	Day 28	Day 35	Day 42	Day 49	Day 56
**4 °C**									
17	17	20	19	13	16	12	16	11	13
150	155	155	157	153	169	126	123	153	147
350	347	336	310	357	357	313	325	323	246
1,500	1,514	1,506	1,419	1,508	1,517	1,221	1,320	1,300	1,342
3,500	3,226	3,396	3,225	3,349	3,439	2,847	2,912	2,940	3,229
5,500	5,379	4,414	5,033	4,687	5,156	4,541	4,349	4,284	4,235
**−20 °C**									
17	17	12	13	13	10	18	11	17	14
150	155	147	157	154	142	128	137	145	142
350	347	351	288	354	356	326	324	261	282
1,500	1,514	1,509	1,459	1,509	1,415	1,271	1,320	1,385	1,388
3,500	3,226	3,158	3,136	3,283	3,191	2,693	2,533	2,703	2,912
5,500	5,379	4,471	3,717	4,779	5,456	4,036	4,176	4,407	3,886
**−80 °C**									
17	17	15	16	16	15	13	13	13	13
150	155	169	160	154	153	140	131	140	162
350	347	347	346	353	340	313	314	337	357
1,500	1,514	1,514	1,503	1,508	1,518	1,277	1,348	1,338	1,631
3,500	3,226	3,325	3,271	3,223	3,534	2,827	2,667	2,407	2,431
5,500	5,379	5,156	5,425	5,331	5,526	4,642	4,242	3,980	4,540

**Notes.**

Results reported in ng/mL; *n* = 1.

* Day 1 samples were not frozen but analyzed immediately after spiking.

**Table 2:** Propofol blood concentrations measured for 56 days at 4 °C, −20 °C, and −80 °C.

Temp	Concentration at different days of storage								
Starting conc.	Day 1^*^	Day 7	Day 14	Day 21	Day 28	Day 35	Day 42	Day 49	Day 56
**4 °C**									
17	18	16	16	21	16	19	17	22	13
150	149	145	145	141	140	145	141	133	126
350	355	345	344	351	351	227	196	220	179
1,500	1,490	1,417	1,314	1,296	1,238	792	913	1,030	825
3,500	3,189	2,409	2,457	2,340	2,426	1,918	1,947	1,817	1,878
5,500	4,558	3,974	4,074	3,878	4,246	3,972	3,431	3,512	2,713
**−20 °C**									
17	18	17	23	26	10	5	5	9	ND
150	149	122	163	163	73	53	35	60	ND
350	355	190	217	254	280	159	115	115	92
1,500	1,490	1,160	1,233	1,217	1,418	1,122	909	1,003	777
3,500	3,189	2,257	3,156	3,139	2,659	1,788	1,262	1,301	1,007
5,500	4,558	4,309	4,184	4,729	4,126	3,455	2,994	3,026	2,404
**−80 °C**									
17	18	16	21	25	19	20	19	21	19
150	149	154	133	1355	142	151	150	158	155
350	355	341	358	345	349	304	357	312	300
1,500	1,490	1,201	1,375	1,297	1,488	1,196	1,198	1,293	1,289
3,500	3,189	2,286	3,122	3,112	2,966	2,430	2,422	2,388	2,225
5,500	4,558	4,092	4,766	4,570	4,528	3,986	3,822	3,870	3,655

**Notes.**

ND, no propofol detected in sample; results reported at ng/mL; *n* = 1.

* Day 1 samples were not frozen but analyzed immediately after spiking.

**Table 3:** Propofol plasma concentrations measured for 56 days at 4 °C, −20 °C, and −80 °C.

Temp	Percentage change in concentration from starting concentration								
Starting conc. (ng/mL)	Day 1	Day 7	Day 14	Day 21	Day 28	Day 35	Day 42	Day 49	Day 56
**4 °C**									
17	0	17.65	11.76	−23.53	−5.88	−29.64	−5.88	−35.29	−23.53
150	3.33	3.33	4.67	2	12.67	−16	−18	2	−2
350	−0.86	−4	−11.43	2	2	−10.62	−7.03	−7.85	−29.71
1,500	0.93	0.40	−5.40	0.53	1.13	−18.62	−12.02	−13.30	−10.53
3,500	−7.83	−2.98	−7.87	−4.31	−1.74	−18.64	−16.80	−16.01	−7.75
5,500	−2.2	−19.75	−8.50	−14.78	−6.25	−17.43	−20.93	−22.12	−23.01
**−20 °C**									
17	0	−29.41	−23.53	−23.53	−41.18	5.88	−35.29	0	−17.65
150	3.33	−2	4.67	2.67	−5.33	−14.67	−8.67	−3.33	−5.33
350	−0.86	0.39	−17.67	1.14	1.17	−6.80	−7.55	−25.43	−19.43
1,500	0.93	0.60	−2.71	0.60	1.20	−15.26	−11.97	−7.66	−7.47
3,500	−7.83	−9.76	−10.40	−6.2	−8.82	−23.06	−27.62	−22.76	−16.80
5,500	−2.20	−18.72	−32.43	−13.11	−0.80	−26.62	−24.07	−19.87	−29.34
**−80 °C**									
17	0	−11.76	−5.88	−5.88	−11.76	−23.53	−23.53	−23.53	−23.53
150	3.33	12.67	6.67	2.67	2	−6.67	−12.67	−6.67	8
350	−0.86	−0.86	−1.14	0.86	−2.86	−10.47	−10.17	−3.80	2
1,500	0.93	0.93	0.20	0.53	1.20	−14.86	−10.16	−10.79	8.73
3,500	−7.83	−5.00	−6.53	−7.91	0.97	−19.22	−23.80	−31.22	−30.54
5,500	−2.20	−6.25	−1.37	−3.08	0.47	−15.60	−22.52	−27.63	−17.45

**Table 4:** Propofol blood concentrations measured for 56 days at 4 °C, −20 °C, and −80 °C.

Temp	Percentage change in concentration from starting concentration								
Starting conc. (ng/mL)	Day 1	Day 7	Day 14	Day 21	Day 28	Day 35	Day 42	Day 49	Day 56
**4 °C**									
17	5.88	−5.88	−5.88	23.53	−5.88	11.76	0	31.03	−24.84
150	−0.67	−3.04	−3.25	−6	−6.52	3.04	−5.70	−11.62	−16.32
350	1.43	−1.43	−1.64	0.29	0.29	−35.25	−44	−37.26	−48.91
1,500	−0.67	−5.50	−12.38	−13.61	−17.45	−47.23	−39.14	−31.32	−45
3,500	−8.89	−31.17	−29.81	−33.15	−30.69	−45.20	−44.36	−48.10	−46.34
5,500	−17.13	−27.13	−25.92	−29.50	−22.80	−27.78	−37.62	−36.14	−50.67
**−20 °C**									
17	5.88	0	35.29	35.29	−42.86	−69.89	−70.59	−46.47	ND
150	−0.67	−18.57	8.67	8.67	−51.45	−64.52	−76.78	−60.23	ND
350	1.34	−45.75	−38.14	−27.43	−19.93	−54.57	−67.14	−67.03	−73.71
1,500	−0.67	−22.68	−17.8	−18.87	−5.48	−25.19	−39.39	−33.14	−48.17
3,500	−8.89	−35.53	−9.82	−10.32	−24.04	−48.91	−63.94	−62.82	−71.24
5,500	−17.12	−21.65	−23.92	−14.02	−24.99	−37.19	−45.57	−44.99	−56.30
**−80 °C**									
17	5.88	−5.88	23.53	47.06	11.76	17.65	11.76	23.53	11.76
150	−0.67	2.67	−11.33	−10	−5.33	0.63	0	5.33	3.33
350	1.34	−2.51	2.29	−1.43	−0.29	−13.11	2	−10.92	−14.29
1,500	−0.67	−19.90	−8.33	−13.53	−0.80	−20.27	−20.17	−13.77	−14.04
3,500	−8.89	−34.69	−10.80	−11.09	−15.25	−30.56	−30.81	−31.76	−36.42
5,500	−17.13	−25.59	−13.35	−16.91	−17.67	−27.53	−30.51	−29.64	−33.55

**Notes.**

ND, no propofol detected in sample.

### Reagents and solutions

Propofol was purchased from US Pharmacopeia (Rockford, MD, USA), and 2,4-ditert-butylphenol (purity 99%) was purchased from Sigma-Aldrich (Saint Louis, MO, USA). All other reagent grade chemicals were purchased from Fisher Scientific (Pittsburg, PA, USA). Water was obtained from a Barnstead (Dubuque, IA, USA) Nanopure Infinity ultrapure water system.

Propofol and 2,4-ditert-butylphenol (internal standard) were dissolved in methanol to produce stock solutions of 100 µg/mL. Dilutions of the propofol stock solution were prepared to produce 1 and 10 µg/mL working stock solutions. Standards were aliquoted into 2 mL vials to prevent evaporation and cross contamination. All solutions were protected from light in bottles wrapped in aluminum foil and stored at −20 °C. Standards were stable for four months at this temperature. Standard curves were prepared by fortifying untreated plasma or blood with propofol to produce a linear concentration range of 5–7,000 ng/mL. The final concentrations used were 5, 10, 25, 50, 100, 250, 500, 1,000, 1,500, 2,000, 2,500, 5,000, and 7,000 ng/mL.

### Sample collection

Fifteen adult, male dogs from the UTCVM research colony were determined to be healthy based on results of physical examination; chemistry panel and history were used. Venous blood collected from the jugular vein of each dog was pooled to minimize the impact of individual differences in blood composition (i.e., hematocrit, total protein concentrations) among dogs. The volume of blood collected from each dog did not exceed 1% of body weight in kilograms. The study was approved by the Institutional Animal Care and Use Committee at the University of Tennessee (Protocol number 2241-0214).

Enough pooled blood was centrifuged to provide plasma for the plasma portion of the study. All blood and plasma samples were immediately spiked with propofol calibration standards (17, 150, 350, 1,500, 3,500, and 5,500 ng/mL) and placed in vials labeled day 1, 7, 14, 21, 28, 35, 42, 49 and 56 for analysis on those dates. Samples were then placed in their respective storage locations (4 °C, −20 °C, −80 °C). Day 1 samples were analyzed immediately after spiking. Only one sample was analyzed for each propofol concentration evaluated (i.e., no replicates).

### Extraction procedure

Canine plasma and blood samples were analyzed using a reverse phase high-performance liquid chromatography (HPLC) method (Yarbrough, Harvey & Cox, 2012). The system consisted of a 2695 separations module, a 2475 fluorescence detector and a computer equipped with Empower software. Propofol was extracted from plasma or blood samples by a liquid extraction method. Briefly, previously frozen samples were thawed and vortexed and 400 µL were transferred to a clean test tube followed by 10 µL of internal standard (100 µg/mL 2,4-ditert-butylphenol). One milliliter of acetonitrile-methanol (75:25) was added and the tubes vortex mixed, covered and placed in the refrigerator for 10 min. The tubes were vortex mixed for 10 s and centrifuged for 15 min at 1,000 × g. The supernatant was removed to a clean tube. The procedure was repeated with an additional 0.5 mL of acetonitrile-methanol, and that supernatant combined. The tubes were centrifuged for 5 min and supernatant was placed in chromatographic vials and 100 µL analyzed.

### Statistical analysis

Actual values and comparative changes in values of the different measured propofol concentrations in blood and plasma samples at varying storage temperatures were used to summarize the effect of storage length on stability of propofol concentration. Percent changes in values at the different days of measurement were also used to evaluate the effect of storage on propofol concentration. Graphical representations of some of the above-assessed effects are presented. Validation parameters for plasma were also calculated.

## Results

Samples were analyzed on days 1, 7, 14, 21, 28, 35, 42, 49, and 56. Concentrations were determined with the exception of two blood samples (17 and 150 ng/mL) on day 56 stored at −20 °C that did not result in any detectable propofol. In both sample media (blood and plasma), samples stored at −20 °C resulted in the lowest propofol measurements of all 3 storage temperatures evaluated. Samples stored at −80 °C had the highest propofol measurements for both media.

Actual values of the different measured propofol concentrations in plasma and in blood at various storage temperatures are reported in Tables 1 and 2 while the percentage of variation of the propofol concentrations compared to the starting concentration are listed in Tables 3 and 4. This information was used to summarize the effect of storage length on the stability of propofol concentration. Due to the large number of graphs generated by this study, graphical representations of the above assessed effect are only presented for two plasma (1,500 and 5,500 ng/mL) and blood (17 and 3,500 ng/mL) concentrations (Figs. 1A, 1B, 2A and 2B).

The same method of analysis that was previously used for propofol in whole blood (Yarbrough, Harvey & Cox, 2012) was used to analyze propofol plasma samples. The method of analysis in plasma produced a linear curve over the same concentration range as blood (5–7,000 ng/mL) with and *r*^2^ greater than 0.999. The intra and inter-assay variability ranged from 2.8%–10% and 3.8%–6.0%, respectively which is very similar to what was observed in the whole blood assay (intra and inter-assay variability ranged from 2.0 to 10% and 0.6 to 11%). The average propofol plasma recovery was 91% while the average recovery for 2,4-ditert-butylphenol was 90%. These recovery values were the same as those for whole blood propofol. The lower limit of quantification was 5 ng/mL, which is the same as the value for whole blood. Calibration curves were constructed each day of analysis for whole blood and plasma.

## Discussion

In order to determine the optimal conditions required for storing propofol samples, blood and plasma were collected from healthy canines and the samples were pooled in order to reduce the effects of inter-individual variability. A method of analysis, which was originally developed, for determination of propofol in blood samples was applied and validated for analysis of plasma samples. To determine whether blood or plasma was the appropriate sample matrix and to determine the effect of storage temperature and duration, blood and plasma samples were spiked with various amounts of propofol that fell within a validated linear concentration range, and stored at three different temperatures (4 °C, −20 °C, −80 °C), and analyzed at various times up to 56 days.

Propofol was detected in blood for all six concentrations for the entire 56 days except for 17 and 150 ng/mL stored at −20 °C on day 56. This could be due to degradation of propofol by enzymes contained in the blood or strong binding to or even penetration into solid blood elements. There was a decrease in propofol concentrations after day 21 for all six concentrations. The impact was greater on samples stored at −20 °C, with an overall average loss of 32% from day 21 to 28. There was some propofol loss for the six different concentrations at 4 °C (9%) and −80 °C (7%) but it is not as dramatic as the loss of propofol in samples stored at −20 °C. The overall loss of propofol from day 1 to day 56 ranged from 37%, 73% and 12% for 4 °C, −20 °C, and −80 °C, respectively. The majority of the samples stored at −80 °C had higher concentrations than those stored at 4 °C and −20 °C which suggests that −80 °C would be an acceptable temperature to store whole blood propofol samples.

There were few differences in concentrations among the storage temperatures for plasma samples. Propofol was detected in plasma for all six concentrations for the 56-day duration. However, after 28 days there was a decrease in propofol concentration for 150, 350, 1,500, 3,500 and 5,500 ng/mL for all three temperatures. The overall average loss was 18%, 15%, and 14% for 4 °C, −20 °C, and −80 °C, respectively. The overall loss of propofol from day 1 to day 56 ranged from 15%, 15% and 8% for 4 °C, −20 °C, and −80 °C, respectively. In general, the concentrations were more consistent in plasma than in blood among the different storage temperatures. However, blood and plasma propofol samples stored at −20 °C had lower concentrations than those stored at 4 °C and −80 °C.

There were differences between plasma and blood propofol concentrations stored at the three different temperatures. There were large differences in concentrations between plasma and blood at 4 °C for 1,500, 3,500 and 5,500 ng/mL and there were also differences in the 350, 1,500, 3,500, and 5,500 ng/mL concentrations stored at −20 °C. Similar differences were also detected at −80 °C, with plasma having greater concentrations at 17, 1,500, 3,500, and 5,500 ng/mL. Although not all plasma propofol concentrations were larger than blood concentrations, many of the individual plasma concentrations were larger than the corresponding blood concentrations, which suggests that plasma would be suitable for propofol analysis.

This method of analysis was applied to samples collected from a study (Reed et al., 2015) conducted previously in this facility in which canines were anesthetized with intravenously administered propofol (6 mg/kg loading dose with mean continuous rate infusion of 0.45 mg/kg/min for 185 ± 32 min) (Table 5). Whole blood and plasma samples were obtained and analyzed within two weeks of collection, after storage at −80 °C. Plasma samples averaged 37% higher concentrations than the same whole blood samples. This is slightly higher than the average difference between the spiked blood and plasma samples from the current stability study. Because the samples were determined with different calibration curves, this may have had an impact on the results; however, any differences should be minor because regression lines and other validation parameters were similar between the two matrices.

**Table 5:** Canine propofol results in blood and plasma samples collected at different time points during general anesthesia induced with an intravenous administration of 6 mg/kg and continuous rate infusion of 0.45 mg/kg/min.

Sample	Blood Propofol ng/mL	Plasma Propofol ng/mL
Mitchell W	1,355	4,143
Mitchell E	3,795	7,207
Mitchell 60	6,194	6,799
Quincy W	1,395	2,388
Quincy E	1,952	3,209
Quincy 60	5,713	7,275
Houston W	2,497	4,073
Houston E	5,717	9,061
Houston 60	3,840	5,658

**Notes.**

Mean anesthesia time (±SD) was 185 ± 32 min. W, samples collected at walking when animals were able to walk upon recovery from anesthesia; E, samples collected at endotracheal extubation time upon recovery from anesthesia; 60, samples collected at 60 min after anesthetic induction.

Although blood has been recommended as the preferred sample for the analysis of propofol concentrations by some authors (Adam et al., 1981; Plummer, 1987; Chan & So, 1990; Zonca et al., 2012; Cattai et al., 2016) the results of the present study indicate a difference between blood and plasma propofol concentrations, at least under the experimental conditions applied in the study. Moreover, the plasma propofol determinations were more consistent than whole blood determinations. Thus, it is important to stress that from an analytical standpoint plasma is the most appropriate sample matrix for propofol analysis. Plasma was also determined to be the sample of choice when compared to blood in a study conducted by Bienert et al. (2005). Plasma was also found to have more consistent results when compared to blood propofol concentrations in mammalian species (Grossherr et al., 2007). Dawidowicz, Fornal & Fijalkowska (2000) also saw a significant decrease in blood propofol concentrations compared to minor changes in plasma samples during 24 days of storage.

## Conclusion

In summary, whole blood and plasma samples containing propofol stored at −80 °C have concentrations as high as or higher than those stored at 4 °C or −20 °C for 56 days; thus, −80 °C is an appropriate temperature for propofol sample storage. While the purpose of the study was not to determine a specific storage time if you apply the FDA Bioanalytical Guidelines for method development that states a value should be within 15% deviation from a standard, then somewhere between 28 and 35 days would be an appropriate storage time for plasma and roughly 21 days for blood. Plasma propofol concentrations were consistently higher than whole blood for all 3 storage temperatures and from Reed et al. (2015) canine study samples. Consequently, plasma is a more suitable sample matrix than blood for propofol analysis providing consistent determinations. The HPLC method that was developed and validated allows for the determination of whole blood and plasma propofol concentrations and is appropriate for use in pharmacokinetic studies.

## Additional Information and Declarations

### Data Availability

The following information was supplied regarding data availability: The raw data is included in the tables in the manuscript.

## References

[ref-1] Adam HK, Douglas EJ, Plummer CF, Cosgrove MB (1981). Estimation of ICI-35868 (Diprivan) in blood by high-performance liquid chromatography, following coupling with Gibbs’ reagent. Journal of Chromatography.

[ref-2] Bienert A, Zaba Z, Dyderski S, Ogrodowiz M, Drobnik L (2005). Long-term stability of propofol in human plasma and blood in different storage conditions. Acta Poloniae Pharmaceutica.

[ref-3] Campos S, Monterio J, Balenzuela B, Goncalinho H, Guedes de Pinho P, Fresco P, Felix L, Antunes L (2016). Evidence of different propofol pharmacokinetics under short and prolonged infusion times in rabbits. Basic & Clinical Pharmacology & Toxicology.

[ref-4] Cattai A, Pilla T, Cagnardi P, Zonca A, Franci P (2016). Evaluation and optimization of propofol pharmacokinetic parameters in cats for target-controlled infusion. Veterinary Record.

[ref-5] Chan K, So APC (1990). The measurement of propofol in human blood samples by Liquid chromatography. Methods and Findings in Experimental and Clinical Pharmacology.

[ref-6] Coetzee JF, Glen JB, Wium CA, Boshoff L (1995). Pharmacokinetic model selection for target controlled infusions of propofol. Assessment of three parameter sets. Anesthesiology.

[ref-7] Cuadrado G, Solares G, Gonzalez S, Sanchez B, Armijo JA (1998). Propofol concentrations in whole blood: influence of anticoagulants and storage time. Methods and Findings in Experimental and Clinical Pharmacology.

[ref-8] Dawidowicz AL, Fornal E, Fijalkowska A (2000). Determining the influence of storage time on the level of propofol in blood samples by means of chromatography. Biomedical Chromatography.

[ref-9] Gomulka P, Fornal E, Berecka B, Szmagara A, Ziomek E (2015). Pharmacokinetics of propofol in rainbow trout following bath exposure. Polish Journal of Veterinary Science.

[ref-10] Grossherr M, Spies E, Scheel A, Hengstenberg A, Gehring H, Dibbelt L (2007). Differences of propofol concentrations in mammalian whole blood and in corresponding plasma samples analyzed by high performance liquid chromatography. Clinical Laboratory.

[ref-11] Lee SH, Park HW, Kim MJ, Noh MH, Yoon HS, Choi BM, Lee EK, Noh GJ (2012). External validation of pharmacokinetic and pharmacodynamic models of microemulsion and long-chain triglyceride emulsion propofol in beagle dogs. Journal of Veterinary Pharmacology and Therapeutics.

[ref-12] Mandsager RE, Clarke Cr, Shawley RV, Hague CM (1995). Effects of chloramphenicol on infusion pharmacokinetics of propofol in greyhounds. American Journal of Veterinary Research.

[ref-13] Mazoit JX, Samili K (1999). Binding of propofol to blood components: implications for pharmacokinetics and for pharmacodynamics. British Journal of Clinical Pharmacology.

[ref-14] Miryabe-Nishiwake T, Masui K, Kaneko A, Nishiwaki K, Nishio T, Kanazawa H (2013). Evaluation of the predictive performance of a pharmacokinetic model for propofol in Japanese macaques (Macaca fuscata fuscata). Journal of Veterinary Pharmacology and Therapeutics.

[ref-15] Plummer GF (1987). Improved method for the determination of propofol in blood by high-performance liquid chromatography with fluorescence detection. Journal of Chromatography.

[ref-16] Reed RA, Seddighi MR, Odoi A, Cox SK, Egger CM, Doherty TJ (2015). Effect of ketamine on the minimum infusion rate of propofol needed to prevent motor movement in dogs. American Journal of Veterinary Research.

[ref-17] Robertson SA, Johnston S, Beemsterboer J (1992). Cardiopulmonary, anesthetic and postanesthetic effects of intravenous infusions of propofol in greyhounds and non-greyhounds. American Journal of Veterinary Research.

[ref-18] Servin F, Desmonts JM, Haberer JE, Cockshott ID, Plummer GF, Farinotti R (1988). Pharmacokinetics and protein binding of propofol in patients with cirrhosis. Anesthesiology.

[ref-19] Yarbrough J, Harvey R, Cox S (2012). Determination of propofol using high performance liquid chromatography in whole blood with fluorescence detection. Journal of Chromatographic Science.

[ref-20] Zonca A, Ravasio G, Gallo M, Montesissa C, Carli S, Villa R, Cagnardi P (2012). Pharmacokinetics of ketamine and propofol combination administered as ketofol via continuous infusion in cats. Journal of Veterinary Pharmacology and Therapeutics.

[ref-21] Zoran Dl, Riedesel DH, Dyer DC (1993). Pharmacokinetics of propofol in mixed breed dogs and greyhounds. American Journal of Veterinary Research.

